# PD-1/PD-L1 blockade in HIV-associated advanced lung cancer: from mechanisms to clinical practice

**DOI:** 10.3389/fimmu.2026.1708278

**Published:** 2026-02-10

**Authors:** Ruifang Chen, Shuai Ren, Haicheng Tang, Qingguo Wu

**Affiliations:** Department of Respiratory and Critical Care Medicine, Shanghai Public Health Clinical Center, Shanghai, China

**Keywords:** HIV, immunotherapy, lung cancer, PD-1 inhibitor, PD-L1 inhibitor

## Abstract

The advent of antiretroviral therapy (ART) has ushered in a remarkable era of prolonged survival for individuals living with Human Immunodeficiency Virus (HIV). Yet this prolonged survival has paradoxically coincided with an increasing burden of non-AIDS-defining malignancies, particularly advanced non-small cell lung cancer. Targeted inhibitors of the programmed cell death protein-1 (PD-1) pathway and its ligand, programmed death-ligand 1 (PD-L1), have now become the cornerstone of modern immunotherapy. Nevertheless, comprehensive studies evaluating the application of these inhibitors in HIV-positive individuals with concurrent lung cancer remain scarce. This review elucidates the dual mechanism of action of PD-1/PD-L1 inhibitors in this patient population: on one hand, blockade of the PD-1/PD-L1 axis enhances anti-tumor activity *in vivo*, while on the other, it reverses latent HIV infection and restores immune function. Current evidence suggests that PD-1/PD-L1 inhibitors demonstrate favorable safety and promising efficacy in ART-controlled HIV patients with lung cancer. However, comprehensive clinical trials remain imperative to validate their long-term outcomes, given the existing limitations of small sample sizes and population heterogeneity in current studies. This article seeks to establish a theoretical foundation for tailoring immunotherapy strategies to this unique patient population.

## 1 Introduction

Programmed cell death protein 1 (PD-1, CD279) is expressed on activated immune cells, including T cells, B lymphocytes, NK cells, macrophages, dendritic cells, and monocytes, with particularly high expression observed in tumor-infiltrating T cells (1). Programmed Cell Death Ligand 1 (PD-L1, also termed CD274 or B7-H1) is a key ligand of PD-1, primarily expressed on antigen-presenting cells and tumor cell surfaces (2). Under physiological conditions, the PD-1/PD-L1 interaction delivers inhibitory signals that suppress T-cell activation, cytokine production, and cytotoxic function. This regulatory mechanism promotes immune tolerance by attenuating CD8^+^ T-cell survival and effector responses (3). Among all immune cells, T cells serve as the most potent effectors in directly eliminating cancer cells. Upon activation by antigen stimulation, the immune system concurrently initiates a negative feedback mechanism to prevent sustained over-activation of T cells and consequent excessive damage to the host. When cancer cells express PD-L1, it can bind to PD-1 on the surface of T cells, thereby triggering this negative feedback pathway. This interaction suppresses anti-tumor immune responses and enables cancer cells to evade immune-mediated killing (3). Blocking PD-1 or PD-L1 therefore enhances the ability of T cells to attack cancer.

Checkpoint molecules—inhibitory receptors expressed on T cells—mediate this immune negative feedback by binding to their corresponding ligands on target cells, thereby inhibiting target cell clearance. In recent years, immune checkpoint inhibitors (ICIs), including specific antibodies targeting PD-1, PD-L1, and cytotoxic T lymphocyte–associated antigen 4 (CTLA-4), have gained prominence for their capacity to overcome T cell tolerance toward tumors. These agents have been adopted as first- or second-line therapies for advanced non-small cell lung cancer (NSCLC) and, compared with chemotherapy or placebo, offering renewed hope for patients with limited therapeutic options (4–8).

Initially, ICIs development centered on PD-1/PD-L1 inhibitor monotherapy as second-line treatment. However, recent advancements have transformed the paradigm, with combination therapies now dominating first-line treatment and immunotherapy being incorporated into earlier clinical models. Currently, nearly all advanced NSCLC patients without targetable oncogenic drivers receive PD-1/PD-L1-based therapy as part of their treatment regimen (9).

Perioperative immunotherapy has emerged as a promising strategy for resectable NSCLC, demonstrating improved survival and enhanced pathological responses compared to conventional approaches (10). Notably, in advanced stage III disease (T4 and/or N2-N3), the integration of neoadjuvant PD-1/PD-L1 blockade with chemotherapy has shown remarkable efficacy, significantly boosting both pathological complete response rates and surgical resectability (11). These seminal studies demonstrate that immunotherapy not only provides viable therapeutic options for operable NSCLC cases, but remarkably extends clinical benefits to borderline resectable and even unresectable patients, thereby expanding the treatment horizon.

HIV infection induces progressive depletion of CD4^+^ T cells, resulting in chronic immunosuppression. Notably, lung cancer has emerged as the predominant cause of cancer-related mortality among HIV-infected individuals, representing 25-40% of non-AIDS-defining malignancies (12). While ICIs targeting PD-1/PD-L1 have revolutionized advanced NSCLC treatment, their application in HIV-infected populations remains clinically problematic. Current trial exclusion criteria for this immunocompromised cohort stem from four principal concerns.

1) Through blockade of immune inhibitory pathways, ICIs enhance antitumor immunity. In people living with HIV (PLWH), immune reconstitution following effective ART can provoke disproportionate inflammatory responses to latent antigens (immune reconstitution inflammatory syndrome, IRIS). Since ICIs further augment immune activation, they markedly increase the likelihood of IRIS onset or worsening in this cohort. 2) Amplified Immune-Related Adverse Events: ICIs exhibit a characteristic class of adverse effects termed immune-related adverse events (irAEs), wherein the hyperactivated immune system may erroneously target the host’s normal tissues, manifesting as pneumonitis, colitis, hepatitis, dermatitis, and other inflammatory conditions. In most PLWH, pre-existing immune dysregulation predisposes them to heightened susceptibility to these irAEs, which often present with greater severity and unpredictable clinical trajectories compared to immunocompetent individuals. 3) The effect of ICIs on immunovirological markers—particularly viral load and CD4^+^ T-cell count—in people living with HIV remains unclear. 4) PLWH represent a vulnerable population, and ethics committees bear the responsibility of safeguarding them from known, potentially fatal risks.

Intriguingly, emerging research suggests that the PD-1/PD-L1 axis may exert a dual immunomodulatory role in HIV-positive patients with lung cancer: (i) Elevated PD-L1 expression within the tumor microenvironment suppresses T-cell-mediated antitumor responses; while (ii) HIV-specific T cells exhibit functional exhaustion due to PD-1 upregulation, suggesting that pathway blockade may concurrently restore antitumor immunity and reverse viral latency (13–17). Nevertheless, immunotherapy for HIV-associated lung cancer remains fraught with challenges, including potential viral reactivation and immune dysregulation. This article comprehensively evaluates the mechanistic rationale, clinical efficacy, and safety profile of PD-1/PD-L1 inhibitors in HIV-positive lung cancer patients, providing evidence-based insights to guide therapeutic decision-making.

## 2 Methods

This study employed a scoping review methodology to systematically search the PubMed database for relevant literature up to July 20, 2024, using keywords such as “HIV”, “Lung cancer”, “SCLC”, “PD-1”, “PD-L1”, and their combinations. The inclusion criteria were: ①Published studies or case reports with full-text availability; ②HIV-positive patients diagnosed with lung cancer; ③Explicit documentation of PD-1/PD-L1 inhibitor therapy. Exclusions comprised comment, conference abstracts, and non-English publications. The review included a total of 32 publications: 11 clinical studies, 15 case reports, and 6 mechanistic reviews. The geographic origins of the clinical studies were France (n=4), the United States (n=3), China, South Korea, and Japan (n=1 each), plus one international multicenter trial. Among the case reports, most were from the United States (n=8) or France (n=5), with single reports from Italy and Qatar. The screening and data extraction were conducted independently by two investigators.

## 3 Results

### 3.1 Role of PD-1/PD-L1 pathway in HIV-positive patients with advanced lung cancer

#### 3.1.1 The immunoregulatory role of the PD-1/PD-L1 axis in cancer pathogenesis

It is well established that activated T cells play a pivotal role in suppressing cancer cells (18). PD-1, predominantly expressed on activated T cells, functions as a negative regulator of T cell activity upon binding to PD-L1 or PD-L2 (Programmed Cell Death Ligand 2) (19). Following tumor antigen recognition by T-cell receptors (TCRs), PD-L1/PD-1 engagement initiates inhibitory signaling pathways that suppress T-cell survival, proliferation, and effector functions while promoting apoptosis of tumor-specific T cells and their differentiation into regulatory T cells (Tregs) (20). T cell exhaustion or dysfunction is widely recognized as a pivotal mechanism undermining T cell responses against tumors and pathogens (21). First identified in chronic infections, this hypofunctional T cell state recapitulates in the tumor microenvironment (TME) (22). Within the TME, exposure to interferon-γ and other cytokines secreted by activated T cells upregulates PD-L1 expression on tumor cells, enabling immune evasion (23, 24). Concurrently, elevated PD-1 expression exacerbates T-cell exhaustion. Thus, therapeutic blockade of the PD-1/PD-L1 axis restores T-cell function, mitigates exhaustion, and enhances anti-tumor immunity (20, 25). The PD-1/PD-L1 axis is dynamically modulated by multiple intracellular signaling pathways in cancer cells, serving as a critical regulator across all stages of tumor development. Importantly, PD-1/PD-L1 engagement activates major oncogenic cascades—particularly the MAPK, PI3K/AKT and JAK-STAT pathways—that coordinately govern essential malignant processes including cellular proliferation, survival and immune escape. These molecular events contribute to tumor progression from initial malignant transformation to metastatic dissemination (26). Recent studies have demonstrated that suppressing the MAPK pathway in lung adenocarcinoma impedes the upregulation of PD-L1 protein, thereby potentially enhancing anti-tumor immune responses and improving the efficacy of immunotherapy (27). Similarly, inhibition of the PI3K-AKT-mTOR pathway has been shown to downregulate PD-L1 expression in PTEN-deficient triple-negative breast cancer cell lines *in vitro (*28). Additionally, the JAK/STAT3 pathway—activated by fibroblast growth factor receptor 2—promotes PD-L1 expression in colorectal cancer mouse models, uncovering a novel regulatory mechanism for immune evasion (29). Moreover, oncogenic pathways such as Wnt drive aberrant proliferation and invasion, while NF-κB enhances tumor survival and aggressiveness by sustaining a pro-inflammatory microenvironment. Concurrently, the Hedgehog pathway further contributes to metastatic progression in advanced cancers. Intriguingly, the PD-1/PD-L1 axis intersects with each of these pathways (26), underscoring its pivotal role across all phases of oncogenesis. These findings collectively position PD-1/PD-L1 as a compelling therapeutic target in precision oncology (26, 30).

#### 3.1.2 The immunomodulatory role of the PD-1/PD-L1 axis in HIV pathogenesis

Iwai et al. first proposed that PD-1 suppresses antiviral immunity in a dependent manner (31). Notably, in HIV-specific T cells, PD-1-mediated upregulation of basic leucine zipper transcription factors impairs T-cell function (32). Further studies confirm that PD-1 expression on virus-specific T cells restricts their antiviral activity (33, 34). Moreover, HIV-specific CD8^+^ T cells exhibit sustained PD-1 expression, which is exacerbated in chronic infection (35). Tregs play critical roles in immune tolerance and homeostasis. Intriguingly, PD-1 is abundantly expressed on Tregs, and flow cytometry analyzes reveal that PD-1 positive T cells harbor elevated levels of viral particles (36, 37). These cells constitute a key viral reservoir in HIV-infected individuals, maintaining persistence even under ART and thereby perpetuating viral latency. It is well established that HIV-positive individuals harbor latently infected cells with integrated viral genomes. These reservoirs evade immune detection, remain refractory to ART, and persist indefinitely during treatment—constituting the primary barrier to achieving an HIV cure (38, 39). Thus, the development of novel interventions against durable HIV reservoirs becomes essential.

**Figure 1:**
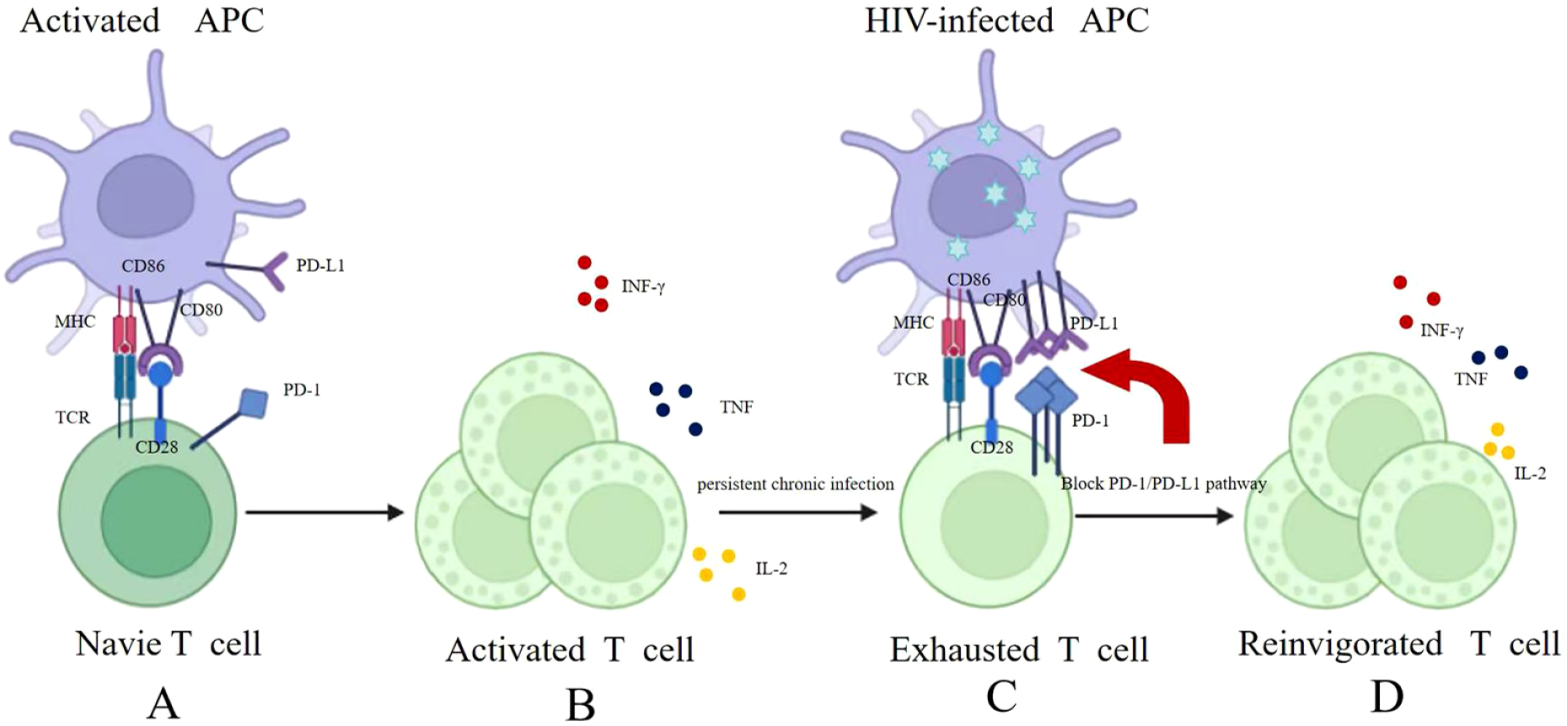
**(A)** Microbial products and cytokines activate antigen presenting cells (APCs), inducing CD80/CD86 expression. Their binding to CD28 promotes immature T-cell expansion and differentiation into effector T cells. **(B)** Effector T cells combat pathogens through cytokine secretion and direct cytotoxicity. **(C)** In chronic HIV infection, T cells become dysfunctional—exhibiting suppressed proliferation, attenuated cytokine secretion, and prominent PD-1 overexpression. **(D)** PD-1/PD-L1 blockade reverses T cell exhaustion, restoring their proliferative capacity and effector functions to enhance anti-HIV immunity.

Previous studies revealed that PD-1 suppresses HIV transcription in CD4^+^ T cells and blocks TCR-mediated viral reactivation in latently infected cells, thereby promoting viral persistence during ART (16), PD-1 blockade reverses HIV latency and, when combined with other therapies, may reduce the viral reservoir (40, 41). In addition, PD-1 inhibitors have been found to reverse the latent infection status of CD4^+^ T cells in HIV infection (16, 40–42) (**Figure 1**) and exhibit favorable safety and tolerability profiles in HIV-positive patients with lung cancer (43, 44).

**Figure 2:**
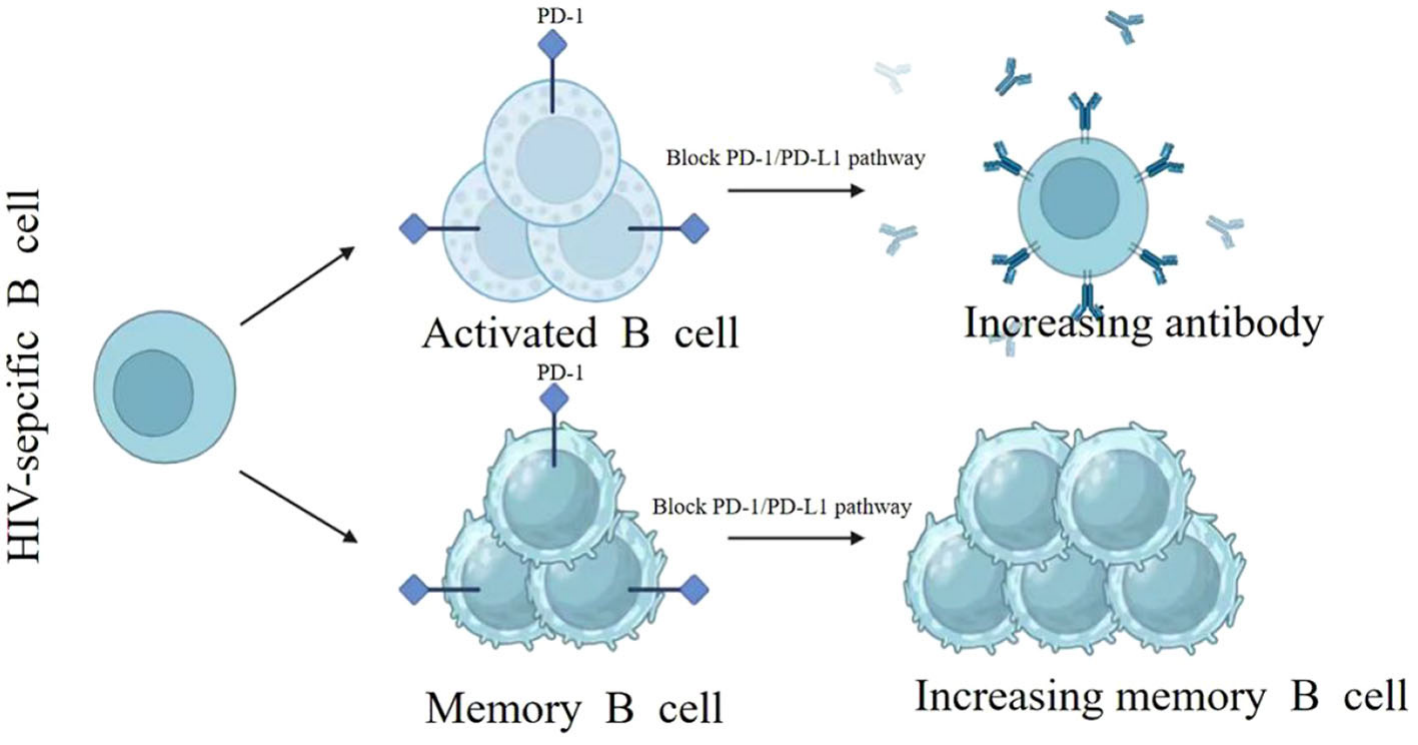
Virus-specific B cells comprise PD-1-expressing effector B cells and memory B cell subsets. Following PD-1 blockade, HIV-activated B cells regain functional capacity to produce virus-specific antibodies. Importantly, PD-1 pathway inhibition enhances memory B cell survival and proliferation, with their exhaustion status directly correlating with disease progression (35).

Moreover, research demonstrates that PD-L1 blockade not only reactivates exhausted CD4^+^ T cells—expanding the pool of HIV-susceptible cells—but also restores dysfunctional B cells to enhance antibody production, offering a potential strategy to curb HIV transmission (45). In HIV-infected patients, disease progression is closely linked to B-cell dysfunction, subset imbalance, and the rapid depletion of activated memory B cells (46). Notably, the PD-1 pathway critically regulates the survival and exhaustion of these memory B cells. Research demonstrates that PD-1 blockade enhances memory B-cell responses in chronic Simian Immunodeficiency Virus (SIV) infection (35, 47) (see **Figure 2**).

Meanwhile, studies suggest that PD-L1 blockade differentially impacts immune effector function depending on subjects’ viremic status. In viremic individuals, PD-L1 inhibition enhanced Treg cell proliferation and viral replication, whereas no significant Treg modulation was observed in those with controlled viremia (48). However, PD-L1 blockade consistently boosted effector T cell expansion across all HIV-infected groups. Since activated CD4^+^ T cells serve as the primary targets for HIV, their activation upregulates PD-1 expression. Upon binding PD-L1, these cells undergo exhaustion. Consequently, PD-L1 blockade may increase the pool of activated T cells—potentially expanding the reservoir of HIV-susceptible targets. Peng X et al. conducted dynamic monitoring of IL-6, HIV DNA, and cell-associated HIV RNA (CA-HIV RNA) in three advanced lung cancer patients with HIV following PD-1 inhibitor treatment. Their findings revealed significant elevations in IL-6 levels, HIV DNA, and CA-HIV RNA within 24 hours post-treatment, with IL-6 showing correlation to HIV RNA levels (44). These findings suggest that PD-1 inhibitor infusion may transiently reactivate the HIV reservoir, potentially due to immune activation of CD4^+^ T cells following PD-1 blockade. However, the study’s limited sample size (n=3) and short follow-up (7 days) constrain broader conclusions. Larger studies are required to evaluate long-term treatment efficacy and viral reservoir control.

Among the strategies targeting latent viral reservoirs, the “shock and kill” approach utilizes latency-reversing agents to reactivate HIV-1 in latently infected cells, thereby unmasking the viral reservoir for immune recognition. Subsequent clearance relies on enhanced immune responses or virus-induced cytotoxicity, ultimately reducing the reservoir burden (49–51). Emerging evidence suggests that combining ICIs with latency-reversing agents—particularly histone deacetylase inhibitors—may offer a promising ART-free strategy for HIV virological control. Mechanistically, latency-reversing agents significantly downregulate PD-L1 expression on cancer cells, synergizing with ICIs to enhance antitumor immunity by facilitating the infiltration of activated T cells into tumor microenvironments (52–54). However, clinical evidence remains scarce regarding the efficacy of PD-1/PD-L1 blockade in achieving ART-free virological control.

Additionally, the *in vitro* model revealed distinct PD-1 and CTLA-4 expression patterns between quiescent and proliferating CD4^+^ T cells. Notably, anti-PD-1 antibodies reversed latency in non-proliferating T cells, while anti-CTLA-4 antibodies targeted proliferating T cells. Thus, the binding of anti-CTLA-4 and anti-PD-1 to distinct T cell subsets may underlie the synergistic therapeutic effect observed with dual PD-1 and CTLA-4 blockade (55, 56).

The dynamic interplay between HIV expansion and immune restriction mechanisms heightens the biological complexity of these therapeutic approaches. Current guidelines recommend immunotherapy exclusively for patients with well-controlled HIV viral loads (45).

### 3.2 Clinical investigation of PD-1/PD-L1 inhibitors in HIV-positive patients with advanced non-small cell lung cancer

Multiple retrospective studies have demonstrated that PD-1 inhibitors (e.g. nivolumab) yield an objective response rate (ORR) of 18%-40% in HIV-positive NSCLC patients, with a median overall survival (OS) of 10.7 months—comparable to outcomes in HIV-negative populations (57, 58). Notably, Guihot et al. observed a marked decline in the HIV viral reservoir in one patient following nivolumab treatment (59), implying a potential synergistic effect between ICIs and ART. However, evidence on first-line ICIs efficacy remains limited. A small-scale study (n=5) reported a median OS of merely 9.8 months, with three patients experiencing rapid disease progression (60), highlighting the marked variability in therapeutic outcomes. Moreover, clinical evidence demonstrates that a significant proportion of HIV-positive NSCLC patients achieve partial or complete remission with PD-1 inhibitor therapy. For instance, Ostios-Garcia et al. observed disease progression in merely two of seven treated patients (58, 61–63). Similarly, a 2019 study involving 21 HIV-positive NSCLC patients—all on ART with undetectable viral loads—revealed that 18% (4/21) attained partial remission and 23% (5/21) maintained stable disease following PD-1 inhibition. However, 55% (12/21) exhibited disease progression at the initial radiological evaluation (57). Emerging evidence demonstrates that the apoptotic protein antagonist inhibitor Debio1143 not only induces tumor cell death but also exhibits synergistic antitumor immunity with PD-1 inhibitors (64). *In vitro* studies reveal its potential to reverse HIV latency while enhancing the efficacy of ICIs in ameliorating immunosuppression and reducing viral load in humanized mouse models (64).

Notably, Byeon et al. reported comparable overall response rates and progression-free survival following PD-1 inhibitor therapy in NSCLC patients, irrespective of specific comorbidities (65). Further supporting these findings, a 2021 Phase II trial observed a 62.5% disease control rate in HIV-positive advanced NSCLC patients treated with nivolumab for eight weeks – an effect independent of baseline CD4^+^ T cell counts – thus reinforcing the clinical rationale for PD-1 blockade in this immunocompromised population (58).

In NSCLC, squamous cell carcinoma represents approximately 20-30% of cases. However, data remain limited regarding HIV-positive patients with this specific histological subtype. A notable case report documented an HIV-positive individual with lung squamous cell carcinoma who initially demonstrated 80-90% tumor regression following chemotherapy (62). Subsequent follow-up revealed lymphadenopathy, with biopsy confirming NSCLC recurrence. Given radiotherapy contraindications, the patient transitioned to nivolumab monotherapy as maintenance treatment. Remarkably, complete remission was achieved without adverse effects, while CD4^+^ T-cell counts remained stable and viral load undetectable throughout treatment (62). In June 2016, another HIV-positive patient was diagnosed with lung squamous cell carcinoma (66). Initial treatment involved first-line chemotherapy and radiotherapy. Upon detecting local progression in 2017, the patient commenced nivolumab maintenance therapy for 28 cycles. Subsequent disease progression necessitated third- and fourth-line chemotherapy, which was discontinued in July 2019. That October, pembrolizumab was administered as a second course of immunotherapy, yielding a favorable response: multiple pulmonary micronodules either resolved or diminished (66). Another HIV-positive patient with lung squamous cell carcinoma received preoperative chemoradiotherapy (67). Despite initial tolerance to nivolumab after lymph node metastasis detection (post-surgery), disease progression emerged post-four cycles, with CD4^+^ T cells dropping from 370 to 130/μl—though HIV-RNA remained undetectable (67). Current clinical data on PD-1/PD-L1 inhibitors for HIV-positive lung squamous cell carcinoma patients remain limited. Large-scale studies are imperative to comprehensively evaluate their efficacy and safety.

A 2023 multicenter retrospective study found no significant difference in the safety and efficacy of PD-1/PD-L1 inhibitors between HIV-positive (n=61) and non-HIV (n=110) patients with advanced NSCLC (68). Furthermore, studies indicate that HIV-positive NSCLC tissues exhibit greater immune cell infiltration and elevated PD-L1 expression compared to those without HIV infection (69). Notably, higher PD-L1 levels correlate with poorer prognosis in HIV-positive patients but not in HIV-negative NSCLC cases (70). These findings suggest that, compared to HIV-negative individuals, PD-1/PD-L1 axis inhibition in HIV-positive NSCLC patients may further suppress anti-tumor immune responses. Consequently, such evidence underscores the potential therapeutic benefit of PD-1/PD-L1 inhibitors for this patient subgroup.

### 3.3 Clinical investigation of PD-1/PD-L1 inhibitors in HIV-positive patients with small cell lung cancer

Small cell lung cancer (SCLC) in HIV-positive patients remains exceptionally rare, with only isolated case reports documented in the medical literature. A 1999 study of 39 HIV-positive lung cancer patients revealed that SCLC accounted for merely 13% of cases, while NSCLC predominated at 78% (71). This scarcity was further evidenced by the Brescia Tropical and Infectious Diseases Clinic cohort (1999–2009), where only one SCLC case was identified among all HIV-associated lung cancer diagnoses (72). A recent case report highlights the clinical challenges: an HIV-positive patient presenting with fever, lymphadenopathy, and back pain was ultimately diagnosed with SCLC (73). Initial treatment with carboplatin, etoposide, and durvalumab showed promising results—rapid symptomatic improvement and no evidence of brain metastasis. However, the patient’s condition abruptly deteriorated during durvalumab maintenance therapy, culminating in death within two months (73). HIV-associated immune alterations may potentially interfere with PD-1 inhibitor activity. Given the paucity of data on PD-1/PD-L1 inhibitors in HIV-associated SCLC (74), robust clinical investigations are urgently needed to elucidate optimal therapeutic strategies for this vulnerable population.

### 3.4 Safety profile of PD-1/PD-L1 inhibitors in HIV-positive patients with advanced lung cancer: focus on adverse reactions

Although PD-1/PD-L1 inhibitors markedly improve cancer patient survival, they can simultaneously impair immune tolerance and elevate the risk of irAEs (75). The most frequent irAEs primarily affect the gastrointestinal tract and skin, manifesting as diarrhea, colitis, pruritus, and rashes. Less common manifestations include endocrine disorders (e.g., thyroid, pituitary, and adrenal dysfunction) as well as hepatic impairment and pneumonitis. For HIV-positive patients with compromised immune function, the likelihood of developing irAEs from ICIs may be substantially elevated. Current evidence indicates that multiple factors—including low baseline CD4^+^ T cell counts, prolonged AIDS duration, prior cancer-related surgery, and positive cytomegalovirus serology at ICI initiation—are significantly associated with severe irAEs (76).

A 2018 study of 7 HIV-positive lung cancer patients receiving PD-1 inhibitors demonstrated favorable overall tolerance, with no grade 3–4 immune-related adverse events or treatment-related deaths observed (63). Supporting these findings, a 2019 JAMA-published trial of 30 pembrolizumab-treated patients with well-controlled HIV (on ART, CD4^+^ T cells >100 cells/μL) reported acceptable safety outcomes (63). Notably, the sole participant with pre-existing KSHV infection succumbed to progressive KSHV-associated lymphoproliferation (77). A 2021 safety study of nivolumab in HIV-positive NSCLC patients revealed that 12 of 16 participants (75%) experienced mild-to-moderate treatment-related adverse events (58). While one case developed severe pruritus, osteomyelitis, and pemphigoid, no opportunistic infections or unexpected immune-related events were observed (58). In addition, recent studies demonstrate that HIV-positive lung cancer patients tolerate immunotherapy well despite lower PD-L1 expression compared to their HIV-negative counterparts (78). Recent multicenter retrospective study by El Zarif et al. demonstrated that HIV-positive patients with CD4^+^ T cell counts <200 cells/μL showed comparable 24-month incidence rates of immune-related adverse events (any grade) to those with CD4^+^ T cell counts >200 cells/μL after ICIs therapy (68). Consistent with these findings, Odeny et al. observed no significant differences in adverse event rates—including grade ≥3 events—between HIV-positive and HIV-negative participants. And the relationship between CD4^+^ T cell counts and adverse reaction incidence remains consistent irrespective of HIV infection status (79). These findings further underscore the importance of abandoning arbitrary CD4^+^ T cell count thresholds when administering immunosuppressants to HIV-positive patients in suitable clinical settings, thereby reducing barriers to treatment given their favorable benefit-risk profile. Additionally, this study suggests that HIV-positive patients with a CD4: CD8 ratio >0.4 exhibit a higher incidence of irAEs (68). This phenomenon may be attributed to an elevated population of circulating CD4^+^ T cells interacting with CD8^+^ T cells (68), this aligns with Lozano et al.’s findings that post-treatment CD4^+^ memory T cells activation and TCR diversity drive irAE pathogenesis (80).

## 4 Discussion

In the field of infectious diseases, immune checkpoints play a critical role in suppressing protective immunity during chronic infections. Notably, the PD-1/PD-L1 axis contributes significantly to T cell exhaustion in HIV-positive patients (17, 81). In advanced HIV infection, the severe depletion of CD4^+^ T cells impairs the immune system’s ability to eliminate virus-infected and cancerous cells. However, blocking the PD-1/PD-L1 interaction has been shown to restore the functionality of virus-specific T cells, offering a potential therapeutic strategy (35, 82). Moreover, PD-1/PD-L1 inhibitors have been widely employed in lung cancer treatment, either as monotherapy maintenance or in combination with chemotherapy. In HIV-positive patients with advanced lung cancer, immune checkpoint upregulation is exacerbated by T cell exhaustion, making combination immunotherapy particularly promising in this population (82).

However, the therapeutic efficacy of PD-1/PD-L1 inhibitors is governed by a constellation of factors: 1) the most significant of these is PD-L1 expression level. While high PD-L1 expression generally predicts greater therapeutic sensitivity, evidence indicates that patients with low PD-L1 expression may still derive clinical benefit (83), particularly when immunotherapy is combined with other modalities such as chemotherapy (84). 2) Equally critical is the TME. An immune-“active” or “inflamed” TME—characterized by abundant tumor-infiltrating lymphocytes (TILs) and elevated inflammatory cytokines—often exhibits up-regulated PD-1/PD-L1 signaling (85) and is associated with improved clinical responses. Several genomic and molecular features also play essential roles. 3) High tumor mutational burden (TMB) generates more neoantigens, enhancing tumor immunogenicity and T-cell recognition, which correlates with better outcomes (86, 87). DNA mismatch repair deficiency (dMMR) leads to high microsatellite instability (MSI-H), thereby increasing TMB. dMMR/MSI-H serves as a robust predictive biomarker for response to immune checkpoint blockade (88). 4) Specific oncogenic driver mutations differentially modulate treatment efficacy. For example, tumors harboring Epidermal Growth Factor Receptor (EGFR) mutations or Anaplastic Lymphoma Kinase (ALK) fusions typically respond poorly to PD-1/PD-L1 inhibitor monotherapy. In contrast, co-mutations in Kirsten rat sarcoma viral oncogene homolog (KRAS) and tumor protein 53 (TP53) are often associated with increased PD-L1 expression and higher TIL density, which may enhance therapeutic sensitivity (89). 5) Finally, growing evidence underscores the considerable influence of the gut microbiota (90, 91). A balanced gut microbiome helps maintain a favorable systemic immune state and can potentiate immunotherapy efficacy. Conversely, dysbiosis or the use of broad-spectrum antibiotics may diminish treatment response.

During treatment with PD-1/PD-L1 inhibitors, the most common adverse events are irAEs, which clinically resemble autoimmune diseases (ADs). Despite this resemblance, patients with a pre-existing history of ADs have been largely excluded from clinical trials due to concerns about exacerbating the underlying AD, increasing the risk of treatment-induced irAEs, and potentially achieving poorer therapeutic outcomes (92).

However, a growing body of recent studies indicates that baseline AD does not statistically significantly worsen clinical outcomes in cancer patients receiving ICIs. Although patients with underlying ADs may experience exacerbations of their baseline condition or be at an elevated risk for other immune-mediated toxicities, these toxicities are mostly reversible with corticosteroid management. Notably, a substantial proportion of these patients do not develop severe irAEs, and no significant association has been consistently found between a history of autoimmune disease and increased mortality (92–94).

However, large-scale clinical trials in HIV-positive lung cancer patients remain scarce, with current evidence limited to small-sample studies or case reports. Beyond the unique challenges of HIV infection, CD4^+^ T cell counts further complicate immunotherapy strategies, inadvertently excluding a high-risk population already burdened by elevated cancer mortality.

This review indicates that PD-1/PD-L1 inhibitors demonstrate acceptable safety profiles in HIV-positive lung cancer patients with well-controlled ART. However, their efficacy appears influenced by tumor heterogeneity, PD-L1 expression levels, and the degree of HIV-associated immune exhaustion. Current limitations include: 1) Limited sample sizes (median n=7) and a predominance of retrospective studies; 2).

Imbalanced histological representation, with most evidence supporting lung adenocarcinoma but lacking data on squamous cell carcinoma and SCLC; 3) Unclear pharmacokinetic interactions between ICIs and antiretroviral drugs. 4) The literature search relied solely on the PubMed database. Although PubMed is a comprehensive and authoritative resource for biomedical literature, this approach inevitably introduces the risk of selection bias, as relevant studies indexed exclusively in other databases may have been omitted. 5) The geographic distribution of the included studies may limit the external validity of our findings. Given that the global HIV epidemic is concentrated primarily in Africa, the Americas, and Southeast Asia, the underrepresentation of studies from these high-burden regions could constrain the generalizability of our conclusions to the global population living with HIV. Therefore, future research should prioritize the conduct of multicenter, prospective trials. These studies should focus on systematically evaluating the risk of viral reactivation, quantifying long-term survival benefits, and validating the predictive value of key biomarkers—such as TMB and TILs.

## 5 Conclusions

Current evidence supports PD-1/PD-L1 inhibitors as a promising therapeutic option for HIV-positive patients with advanced lung cancer, particularly those maintaining ART-controlled viral loads and CD4^+^ T cell counts >200/μL. Their dual mechanism - simultaneously activating anti-tumor immunity and potentially reversing HIV latency - offers novel insights for achieving HIV “functional cure.” However, clinicians should remain vigilant about transient viral reservoir activation during early treatment phases, necessitating optimized therapeutic timing through multidisciplinary coordination. Future investigations should integrate tumor genomic profiling with HIV reservoir monitoring to advance personalized immunotherapy strategies.
